# Effects of Soil Fertilization on Terpenoids and Other Carbon-Based Secondary Metabolites in *Rosmarinus officinalis* Plants: A Comparative Study

**DOI:** 10.3390/plants9070830

**Published:** 2020-07-02

**Authors:** Maria Ángeles Bustamante, Marco Michelozzi, Anna Barra Caracciolo, Paola Grenni, Janine Verbokkem, Peter Geerdink, Carl Safi, Isabel Nogues

**Affiliations:** 1 Department of Agrochemistry and Environment, Miguel Hernandez University, EPS-Orihuela, ctra. Beniel km 3.2, 03312 Orihuela, Spain; marian.bustamante@umh.es; 2 Institute of Biosciences and Bioresources, National Research Council, via Madonna del Piano 10, 50019 Sesto Fiorentino, Florence, Italy; marco.michelozzi@cnr.it; 3 Water Research Institute, National Research Council, Via Salaria km 29.300, 00015 Monterotondo, Rome, Italy; barracaracciolo@irsa.cnr.it (A.B.C.); grenni@irsa.cnr.it (P.G.); 4 Wageningen Food & Biobased Research, Wageningen University and Research, P.O. Box 17, 6700 AA Wageningen, The Netherlands; Janine.verbokkem@wur.nl (J.V.); peter.geerdink@wur.nl (P.G.); carl.safi@wur.nl (C.S.); 5 Research Institute of Terrestrial Ecosystems, National Research Council, Via Salaria km 29.300, 00015 Monterotondo, Rome, Italy

**Keywords:** compost, monoterpenes, sesquiterpenes, leaf nitrogen, leaf phosphorus, phenolic compounds, flavonoids, structural carbohydrates

## Abstract

*Rosmarinus officinalis* is an evergreen aromatic plant with important commercial interest as it contains numerous essential oils (composed of terpenoid compounds) and phenolic constituents (natural antioxidant compounds). This work aims at evaluating the concomitant effects of different inorganic and organic fertilization treatments and the subsequent increases in soil nutrient availability on terpenoids and other carbon-based secondary metabolites, e.g., flavonoids and phenolic compounds, in *Rosmarinus officinalis* leaves. The results showed that, as expected, the structural carbohydrate content (lignocellulosic compounds) in stems was higher in fertilized plants than in controls. Additionally, positive correlations were observed of the absolute amounts of total terpenoids and some single terpenoid compounds with N or P contents in leaves. On the contrary, the phenolic and flavonoid concentrations in all the rosemary plant parts were lower with the fertilization treatments. Indeed, negative correlations between the phenolic compounds (and flavonoids) and N in rosemary leaves were also found. Overall, the results suggest that the terpenoid production’s response to fertilization was due to N, which is essential for protein synthesis and terpene synthase activity, and to P, which is necessary for the synthesis of both terpenoid precursors and ATP and NADPH, also needed for terpenoid synthesis. On the other hand, the basis for the fertilization’s effects on the production of phenolic compounds is the direct nitrogen trade-off between growth and the shikimic acid pathway by which phenolics compounds are synthesized.

## 1. Introduction

Within the Biogenic Volatile Organic Compounds (BVOCs) synthesized and emitted by plants, volatile terpenoids constitute the majority group. Terpenoid emission is a crucial defense mechanism against abiotic [1] and biotic stresses [2] and is able to mediate ecological interactions with the biotic environment [3,4,5,6,7]. In addition, terpenoids participate in the protection of leaves against thermal and oxidative stresses [8,9], probably by the enhancement of membrane stability and the scavenging of reactive oxygen species [10].

Some plant species store terpenoids in specialized structures (storing plants), such as resin ducts, resin blisters, leaf storage cavities or glandular trichomes [11,12,13]; the last of these is the case for the perennial plant species of the Mediterranean area *Rosmarinus officinalis* [13]. These permanent BVOC reservoirs, principally filled with monoterpenoids and sesquiterpenoids, constitute a potential source of BVOC emissions. Moreover, these terpenoid pools, together with other secondary metabolites (phenolics and flavonoids), in aromatic plants (such as *Rosmarinus officinalis*) represent a source of biologically active compounds for the food, pharmaceutical and chemical industries [12,14]. In addition, there is a growing interest in the use of essential oils as possible antimicrobial agents with a low risk of microbial resistance development, offering an alternative to synthetic antibiotics [15].

The main variables controlling the production and emission of terpenoids that are not kept in permanent pools are light (photosynthetically active radiation) and temperature [16,17,18], which affect substrate availability and enzyme activities, respectively [19]. Nevertheless, additional environmental factors (e.g., CO_2_ and ozone levels, seasonality, drought and mechanical stresses) have also been found to affect the emissions of terpenoids [1,20,21,22,23,24,25,26,27]. In the case of “stored terpenoids”, their production seems to also be related to carbon substrate availability, to reductive (NADPH) and energetic equivalents (ATP) and to terpene synthase activity [28,29,30,31,32]. Although changes in storage pools have also been observed as a consequence of environmental constraints (e.g., warming and drought) or stress-related processes [33,34], the relative contents of monoterpenes (monoterpene profiles) in healthy and mature tissues seem to be little affected by abiotic factors and are under strong genetic control [35,36,37,38]. There is much scientific literature concerning the use of terpenoids, particularly monoterpenes, in chemosystematic classification for conifer and aromatic plants. Monoterpene profiles have demonstrated their utility as biochemical markers in distinguishing plant species, hybrids and populations within a group of species, families and clones [39,40,41].

On the other hand, soils in the Mediterranean area often suffer from organic matter and nutrient deficiencies [42], mainly of macronutrients that are essential in the soil–plant system. The presence of unfavorable weather conditions combined with intensive agricultural management constitutes some of the principal reasons for the loss of organic matter in soils [43] and, consequently, of soil degradation, with adverse impacts on plant growth and yield [44]. Within this context, compost incorporation into degraded soils constitutes an affordable green approach to enhancing soil quality, and augmenting the concentrations of organic matter and nutrients such as N and P in the soil [45,46]. Compost can affect plant terpenoid contents in “storing species” since N, which is supplied via compost amendments [47], can promote the electron transport rate and leaf photosynthesis, which provide ATP to meet requirements and make carbon substrates available for terpenoid synthesis [48,49]. Moreover, according to the carbon–nutrient balance hypothesis (CNBH) and the growth–differentiation balance hypothesis (GDBH), there is a relationship between the availability of carbon and nitrogen in the environment and secondary metabolite production. The CNBH presumes a decrease in the concentration of carbon-based secondary metabolites (e.g., phenolics and terpenoids) with an increase in the availability of nutrients [50,51], whereas the GDBH predicts a trade-off between the costs of secondary metabolites and the demand for photosynthates by growth [51,52] under moderate-to-high nutrient availability conditions. However, it has not, to date, been clear how soil nutrients affect leaf terpenoid storage in woody species, and divergent results have been reported in this regard. For example, following N fertilization, the terpenoid contents in *Pinus sylvestris* needles were found to increase [53] but also decrease [54] or remain unchanged [55]. Ormeño et al. [56] reported that the total monoterpene and total sesquiterpene contents of *Pinus halepensis* were significantly and positively correlated with soil N, whereas Blanch et al. [57] reported that fertilization treatments did not significantly affect terpene concentrations in *P. halepensis*. Ormeño et al. [56] also reported a lack of correlation between total leaf terpenoids in *R. officinalis* and soil N and P. Finally, there have been few studies [58] evaluating simultaneous terpenoid and phenolic compound production under the same nutrient conditions.

The results reported here relate to a *Rosmarinus officinalis* plant experiment in which three different fertilizations (two composts derived from cattle or pig anaerobic digestate, respectively, and an inorganic fertilizer) were compared and terpenoids and other carbon-based secondary metabolites (phenolics and flavonoids) were quantified. The aim was to gain insight into and compare the mechanisms controlling the synthesis/production of terpenoids and other types of secondary metabolites under the same nutrient conditions, considering the CNBH and GDBH hypotheses. In particular, the terpenoid contents, phenolic compounds, flavonoids and structural carbohydrates were analyzed in *R. officinalis* grown in a soil with a low organic carbon content and to which two different fertilizers in the form of livestock anaerobic digestates or an inorganic fertilizer were added; the results were compared with those with a non-treated soil.

## 2. Material and Methods

### 2.1. Characteristics of the Soil and of the Composts Used as Organic Amendments

In this study, the soil used came from an abandoned agricultural site located at Montelibretti (Rome, Italy). The details concerning soil sampling and characteristics are detailed elsewhere (Barra Caraccciolo et al., 2015). The composts used (CS, cattle slurry and PS, pig slurry) were prepared using the solid fraction of the digestates obtained after the anaerobic digestion of cattle and pig slurry, respectively, For this, these wastes were mixed with vine shoot pruning in the proportion 75:25 by dry weight. The details concerning the composting process are described in previous work [46,59]. The composts showed high total N contents (29.0 g kg^−1^ and 30.3 g kg^−1^ for CS and PS, respectively) and a good degree of maturity for their use as organic amendments [46,59].

### 2.2. Experimental Design

The study was conducted in a polycarbonate heated greenhouse located in the experimental field of the Terrestrial Ecosystems Research Institute (IRET-CNR) (42°06′12″ N 12°38′53″ E, elevation 227 m a.s.l., Montelibretti, Rome, Italy). For the experimental set-up, 1 kg of soil thoroughly mixed with the composts PS or CS was put into polyethylene pots. Two doses were used (on a fresh weight basis): a) Low dose (Low), adding 11.54 g of compost per kg of soil (corresponding to a dose of 30 t ha^−1^), and b) high dose (High), adding 23.08 g of compost per kg of soil (equivalent to a dose of 60 t ha^−1^). An inorganic fertilization (InOrg) was set up, treating the soil with an inorganic NPK fertilizer in a proportion of 100:60:73. This was obtained by adding the commercial fertilizer Nitrophoska Top 20 (NPK = 20:5:10; 192 mg kg^−1^ soil) and monopotassium phosphate (NPK = 0:52:34; 26 mg kg^−1^). Soil without fertilization was set up as a control treatment. In the experimental design were established three replicates per treatment (18 experimental pots). Genetically identical rooted cuttings of rosemary were planted, one in each pot filled with the corresponding soil treatment. The pots were distributed in a randomized complete block design inside the greenhouse and kept under controlled temperature (25 °C) conditions. The pots were watered regularly, and the soil was maintained gravimetrically at 50% of its field capacity throughout the experiment. All the analyses were performed at the end of the experimental period. The rosemary leaves were collected and divided into two groups. One was frozen with liquid nitrogen and kept at −80 °C until the analysis of the stored terpenoids. The other was dried in a forced air oven at 60 °C for 72 h. Stems were also collected and dried. The fresh weight to dry weight ratio was calculated. The dried samples were then ground to a mean size of 0.5 mm for the analysis of foliar and stem nitrogen, phosphorus, potassium, phenolic compound, flavonoid and structural carbohydrate content.

### 2.3. Nitrogen, Phosphorus and Potassium Contents in Leaves and Stems

The N in tissue samples (leaves and stems) was determined in an automatic elemental microanalyzer (EuroVector Elemental Analyser, Milan, Italy) [60] and expressed as %N. The total concentrations of P and K in leaves and stems were evaluated in the extract following HNO_3_–HClO_4_ digestion. K was determined by atomic absorption spectrophotometry (Analyst 300; Perkin Elmer, Rodgau, Germany) [61] and expressed as g kg^−1^ (dry matter, d.m.). The P concentration was determined calorimetrically using the vanadomolybdate procedure and expressed as g kg^−1^ (d.m.) [62]. The analyses were done with three replicates per treatment.

### 2.4. Terpenoid Analysis

The terpenoids were analyzed in frozen leaves (three replicates per treatment) for each rosemary plant and treatment. The composition of the terpenoid fraction of the rosemary leaves was also determined. Foliar tissues (0.2 g) were ground in liquid nitrogen and extracted with 2.0 mL of N-pentane with tridecane as an internal standard; each sample was filtered, and a 0.5 mL volume was injected in a GC in splitter mode (a 20:1 split ratio; see below for details). The analyses were performed with an AutoSystem XL GC (PerkinElmer) equipped with an automatic sampler for liquid sample injections and with the chromatography software TotalChrom version 6.2.0.0.0:B27. The separation of the different enantiomeric monoterpenes was performed on an Elite-Betacydex Betacyclodextrin capillary column (PerkinElmer), 30 m long and 0.25 mm in diameter. The analysis was carried out using the following instrumental conditions: H_2_ (carrier gas), 2.0 mL min^–1^; injector temperature, 230 °C; detector temperature, 250 °C. The oven temperature program started at 40 °C for 3 min and increased to 200 °C at a rate of 1 °C min^−1^; the final temperature of 200° C was maintained for 10 min. The terpenoids were identified by the comparison of the retention times with those of standards under the same conditions. High purity components were obtained from Sigma-Aldrich S.r.l. (Milan, Italy) and Acros, Geel (Belgium). The absolute amount of each terpenoid (terpenoid concentration) was determined by comparison with the tridecane used as the internal standard and expressed as mg g^−1^ dry weight (d.w.). The leaf dry mass weight was determined after drying the residual vegetal material at 60 °C for 72 h. The relative amount (proportion or percentage) of each compound was expressed as a percentage of the total terpenoids (terpene profiles).

### 2.5. Total Phenolics and Flavonoids

Total phenolic compounds and flavonoids were analyzed (three replicates per treatment) in the leaves, stems and roots for each rosemary plant condition. The extraction of total phenolic compounds and flavonoids was performed from 200 mg of plant material with 80% methanol (1.5 mL) for 3 min in an ultrasonic bath. The extraction was repeated twice.

The total phenolic compounds were measured with the Folin–Ciocalteu reagent [63]. Gallic acid was used as the standard, and the total phenolic compounds were reported as mg of gallic acid equivalents (GAE) per g of dry weight.

The total flavonoid content was determined using the aluminum chloride method as described by Chang et al. [64]. The absorbance was read at the 415 nm wavelength. Solutions of quercetin were used to obtain a standard curve. The total flavonoid content was expressed as mg of total quercetin equivalents per g of dry weight.

### 2.6. Structural Carbohydrates

Polysaccharides from the lignocellulosic plant material, stems and leaves (c.a. 37 mg) (three replicates per treatment) were hydrolyzed following a two-step procedure: 1) strong sulfuric acid pre-hydrolysis (72%) at 30 °C followed by 2) a hydrolysis at 95 °C after diluting the primary hydrolysis slurry. Saccharides were determined by HPAE (High-Performance Anion-Exchange) chromatography with pulsed amperometric detection [65].

### 2.7. Statistical Analysis

The relative contents and the square root and arcsine transformed percentage data of the terpenoids did not meet the requirements of normality and homogeneity of variances evaluated by means of the Kolmogorov–Smirnov and Levene’s tests, respectively. Consequently, the statistical analysis was performed using the non-parametric Kruskal–Wallis rank-sum test.

Analysis of variance (ANOVA) was performed with either terpenoid concentrations, phenolic contents, flavonoid contents or carbohydrate contents as the dependent variable and the organic fertilization as the independent factor. The Fisher post-hoc test was used to investigate the significance of different groups of means, considering a probability level of *p* < 0.05.

Analyses were conducted of the correlation between the leaf total terpenoid concentration (Y variable) and leaf N (X variable) or P (X variable); between the leaf single terpenoid concentration (Y variable) and leaf N (X variable) or P (X variable); between the leaf phenolic compounds or leaf flavonoids (Y variable) and leaf N (X variable); and between the leaf total structural carbohydrates (Y variable) and leaf N (X variable). All the statistical analyses were conducted using SIGMASTAT and the Systat 13.0 software (Systat Software Inc., Richmond, CA, USA).

## 3. Results

### 3.1. Total N, P and K in Rosmarinus Leaves and Stems

The concentrations of total N, P and K in the leaves and stems of the rosemary plants grown in the fertilized and control soils are shown in Table 1.

In general, the organic amendments produced an increase in the amount of leaf and stem N compared to those in the control, although this increase was significant only for the PS_High_ treatment. Surprisingly, the InOrg treatment did not induce any increment in leaf or stem N compared to that in the control. Leaf P turned out to be significantly higher (*p* < 0.05) only in the PS_High_ treatment, whereas we did not observe any difference in stem P content among the treatments. As regards the leaf K concentration, this turned out to be significantly higher in the CS_Low_ leaves than in the control leaves. On the contrary, leaf K contents were significantly lower in the CS_Low_ leaves than in the control ones, whereas the PS_High_ leaves had the highest K concentration.

### 3.2. Terpenoids

In total, twenty-eight terpenoid compounds were found in the rosemary foliar tissues. The mean relative abundance ± standard error of these compounds is shown in Table 2.

Twenty-three compounds were monoterpenoids, of which seventeen (α-pinene, camphene, sabinene, β-pinene, myrcene, limonene, p-cymene, cineole, γ-terpinene, terpinolene, linalool, camphor, terpinen-4-ol, borneol, bornyl acetate, verbenone and geraniol) were present in substantial amounts, while six (δ-3-carene, α-terpineol, geranyl acetate, carvone, thymol, and carvacrol) were detected in very small amounts or traces. The other compounds were two sesquiterpenoids, β-caryophyllene and caryophyllene oxide; and three unknown compounds.

The most abundant monoterpenes were α-pinene (36.4 ± 1.1), verbenone (13.2 ± 2.5), camphor (9.1 ± 0.5), camphene (8.2 ± 0.4) and p-cymene (8.0 ± 0.3). Other terpenoids were the monoterpenes β-pinene, limonene, myrcene, terpinene, terpinolene, sabinene, cineole, linalool and terpineol and the sesquiterpenes β-caryophyllene and caryophyllene oxide. Kruskal–Wallis ANOVA results showed that variations in the relative contents of terpenoids were not significantly affected by the different fertilizing treatments.

**Figure 1:**
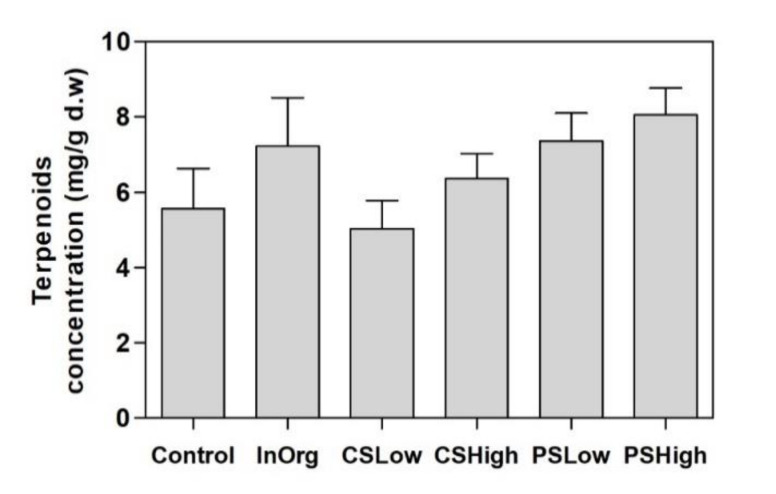
Total terpenoid leaf concentrations (mg g^−1^ d.m.) in *Rosmarinus officinalis* plants in the various fertilization treatment groups: Control, InOrg, CS_Low_, CS_High_, PS_Low_ and PS_High_. Vertical bars indicate standard errors of the mean (*n* = 3).

**Figure 2:**
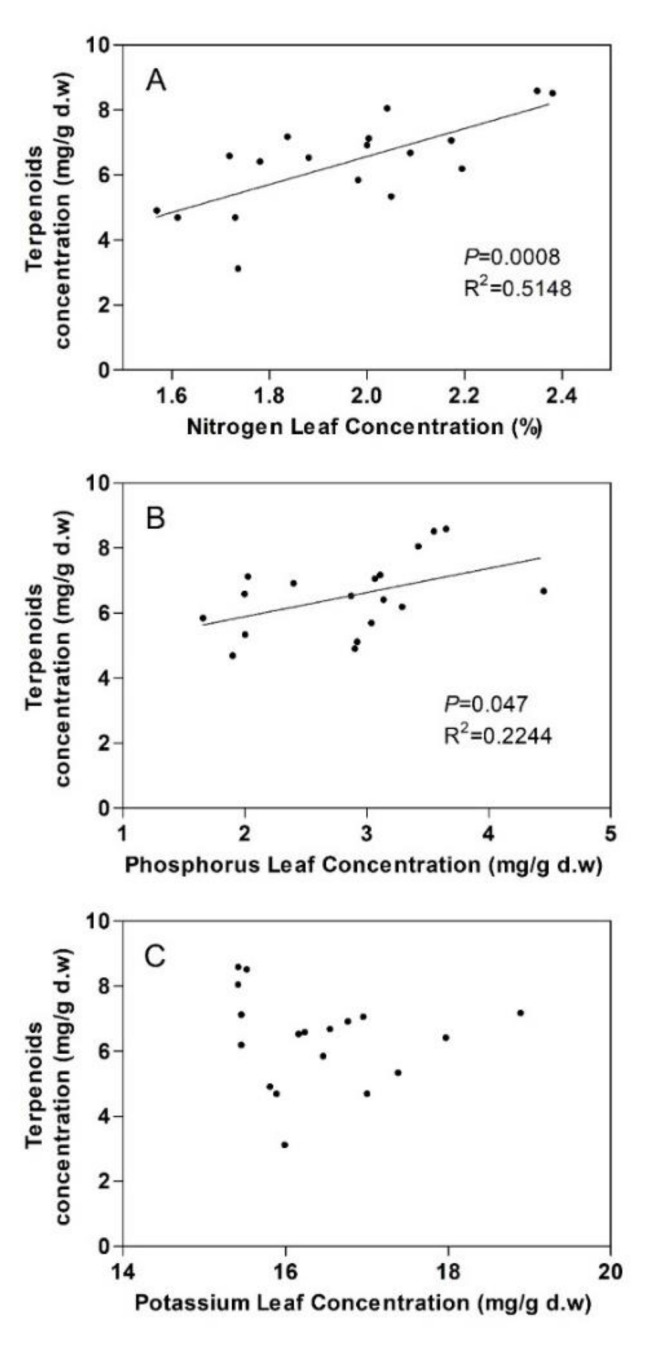
Correlation of total terpenoid leaf concentrations with (**A**) nitrogen leaf concentrations, (**B**) phosphorus leaf concentrations and (**C**) potassium leaf concentrations, for *Rosmarinus officinalis* plants (*n* = 18).

Although the total terpenoid contents did not show significant differences among the fertilizing treatments (Figure 1), positive correlations were found between the total terpenoid concentrations and leaf N (Figure 2A) (*p* < 0.001) and between total terpenoid concentrations and leaf P (Figure 2B) (*p* < 0.05).

**Figure 3:**
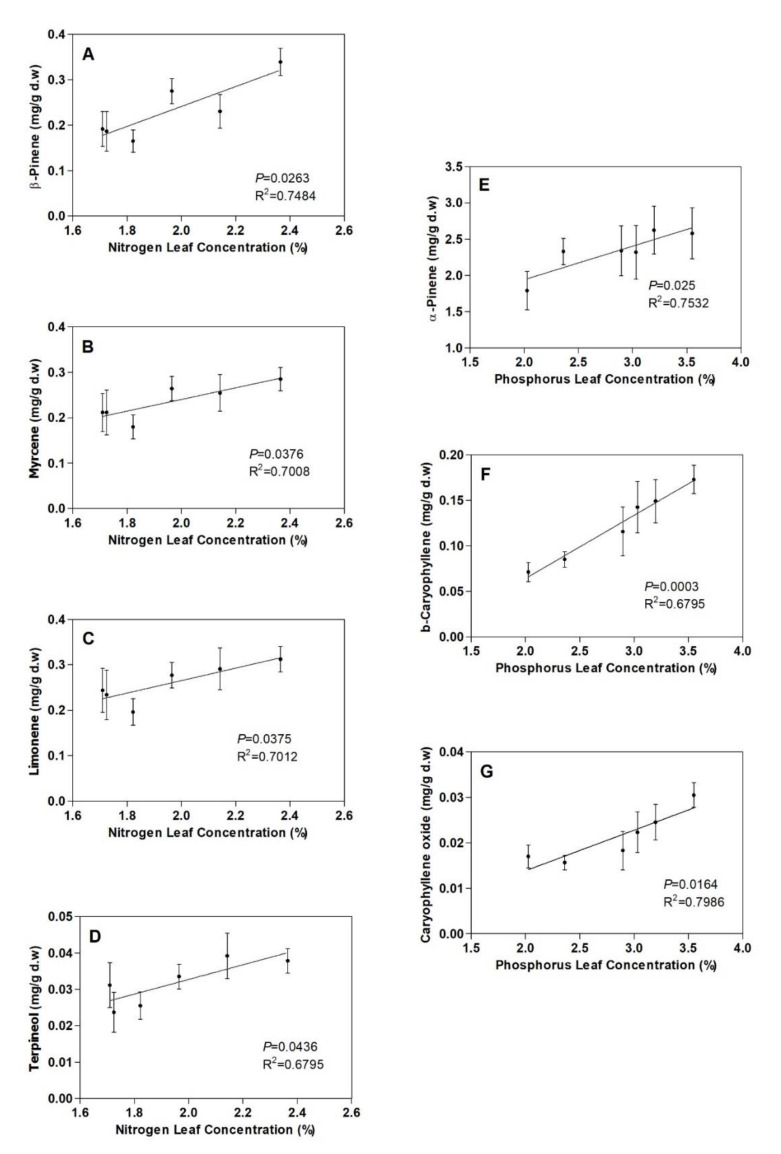
Correlation of leaf (**A**) β-pinene, (**B**) myrcene, (**C**) limonene and (**D**) terpineol concentrations with nitrogen leaf concentrations; correlation of leaf (**E**) β-pinene, (**F**) β-caryophyllene and (**G**) caryophyllene oxide concentrations with phosphorus leaf concentrations. Vertical bars indicate standard errors of the mean (*n* = 3).

In addition, some of the individual monoterpenes increased with leaf N—β-pinene, myrcene, limonene and terpineol (*p* < 0.05) (Figure 3)—whereas α-pinene and the sesquiterpenes β-caryophyllene and caryophyllene oxide increased significantly with leaf P (*p* < 0.05) (Figure 3). No significant correlation between the total terpenoid concentration and K was found (Figure 2C).

### 3.3. Total Phenolic and Flavonoid Content

**Figure 4:**
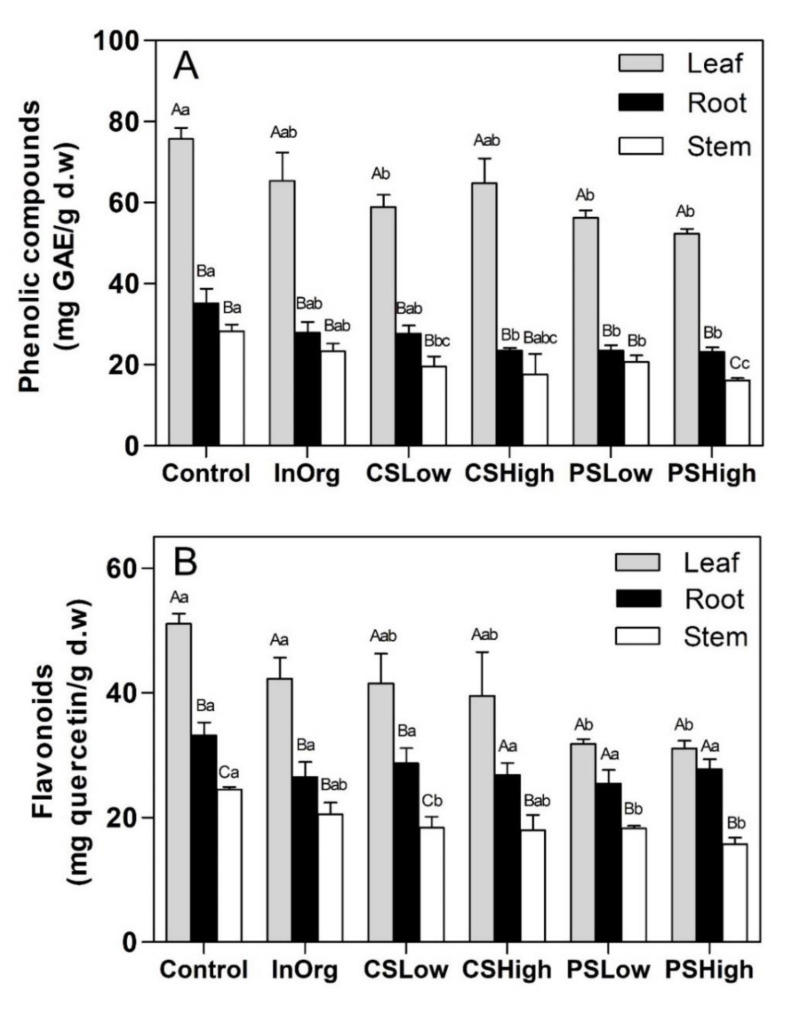
(**A**) Total phenolics and (**B**) total flavonoids in leaves, roots and stems of *Rosmarinus officinalis* plants in the various fertilization treatment groups: InOrg, CS_Low_, CS_High_, PS_Low_ and PS_High_. Vertical bars indicate standard errors of the mean (*n* = 3). Different lowercase letters indicate significant differences among fertilization treatments (*p* < 0.05). Different capital letters indicate significant differences among plant parts (*p* < 0.05).

The phenolic compounds and flavonoids were higher in leaves than in roots and stems (Figure 4A,B) (*p* < 0.001). Moreover, both compounds changed significantly with the fertilizing treatments in each plant part (*p* < 0.05) with the exception of flavonoids in the roots.

In general, phenolic compounds were lower in plants grown on the organic amendments (compost) than in those grown in the control soil (Figure 4A), although these differences were significant only in the following cases: leaf phenolics; CS_High_, PS_Low_ and PS_High_ root phenolics, and PS_High_ stem phenolics (*p* < 0.05). As regards flavonoids, they were significant lower in PS_Low_ and PS_High_ than in control leaves and stems.

**Figure 5:**
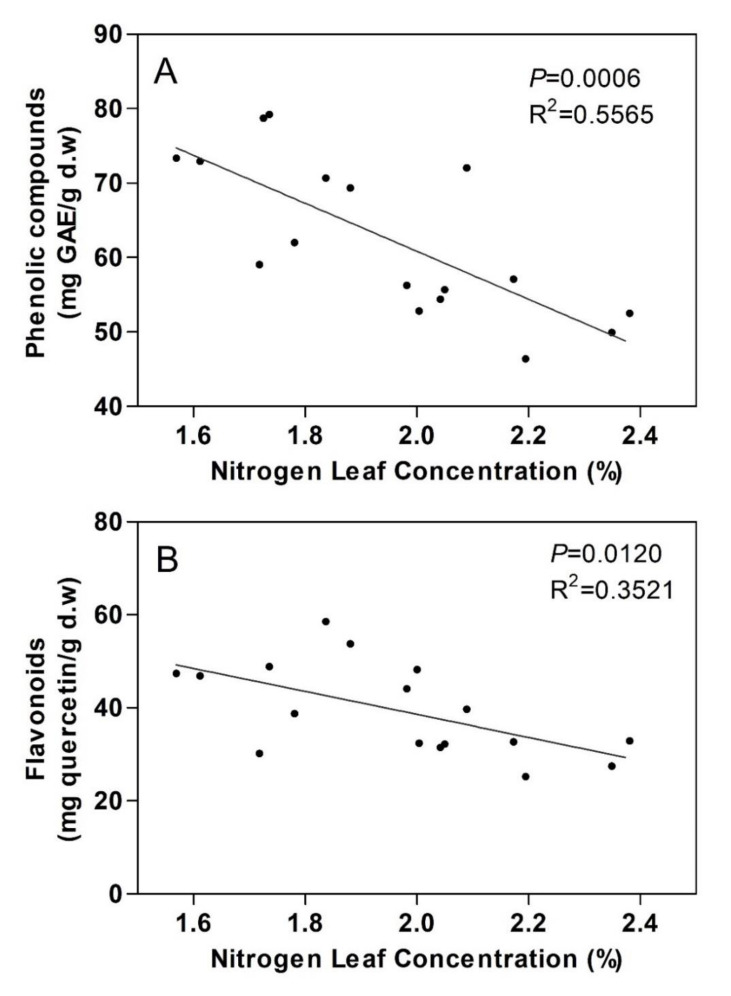
Correlation of (**A**) leaf phenolic compound concentrations and (**B**) leaf flavonoid concentrations with nitrogen leaf concentrations (*n* = 18).

A negative correlation was found between total phenolic compound concentrations and leaf N (Figure 5A) (*p* < 0.001) and between total flavonoid concentrations and leaf N (*p* < 0.05) (Figure 5B). However, no significant correlations were found between leaf phenolics and leaf K, leaf phenolics and leaf P, leaf flavonoids and leaf K and leaf flavonoids and leaf P (data not shown).

### 3.4. Structural Carbohydrates

**Figure 6:**
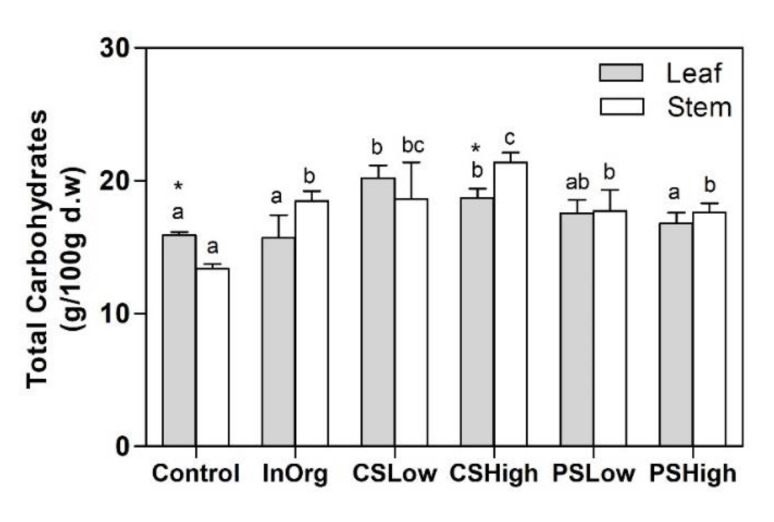
Structural carbohydrates in leaves and stems of *Rosmarinus officinalis* plants for the fertilizing treatments: Control, InOrg, CSLow, CSHigh, PSLow and PSHigh. Vertical bars indicate standard errors of the means (*n* = 3). Different lowercase letters indicate significant differences among fertilization treatments (*p* < 0.05). Asterisks indicate significant differences among plant parts (*p* < 0.05).

The hydrolysis of the polysaccharides from the lignocellulosic material, Rosmarinus stems and leaves, led to the production of glucose, galactose, arabinose, mannose, xylose, fucose and rhamnose (Table 3). The total saccharides varied depending on the fertilization treatment (*p* < 0.05) (Figure 6). In general, they were higher in the plants grown in organic amendments than in those in the control soil; moreover, they were significantly higher in the case of the soil amended with the compost derived from the cattle anaerobic digestate at both doses (CS_Low_ and CS_High_).

**Figure 7:**
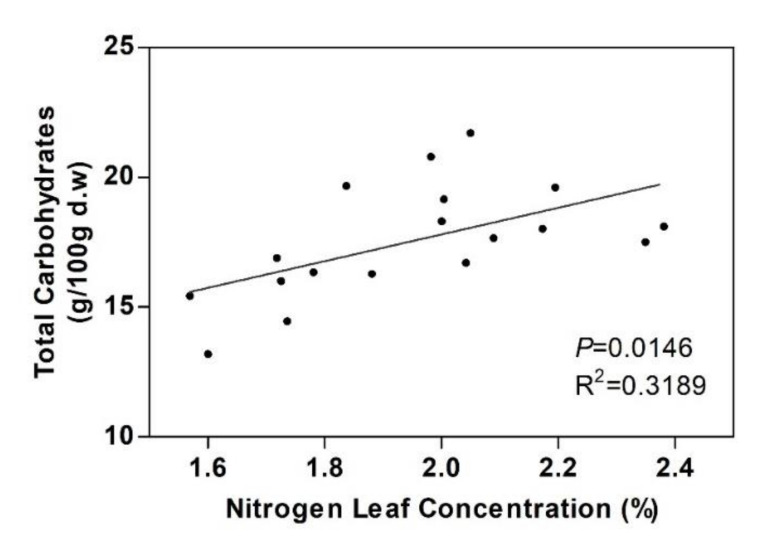
Correlation of leaf total sugars from structural carbohydrates with nitrogen leaf concentrations (*n* = 18).

Interestingly, a positive correlation between thetotal saccharides and leaf N was found (*p* < 0.05) (Figure 7).

## 4. Discussion

The leaf total terpenoid concentrations between 5 and 12 mg g^−1^ d.w., found in this experiment, were in the range of those reported in other studies for rosemary plants [56,66]. In our study, the most abundant terpenoids were α-pinene, verbenone and camphor. However, other studies showed other terpenoids as the most abundant. For instance, Ormeño et al. [56] found camphor, cineole and α-pinene, whereas Llusià et al. [66] identified α-pinene, camphene and β-pinene as the most abundant in *R. officinalis* leaves. These results may reflect differences in the chemotypes of *R. officinalis* [67].

The main goals of this paper were 1) to evaluate the effects of nutrient availability on terpenoid content in *Rosmarinus officinalis* plants and 2) to compare the effects of nutrient availability on terpenoid content with the effects on primary (structural carbohydrates) and other carbon-based secondary metabolites (phenolic compounds and flavonoids). According to the carbon–nutrient balance (CNBH) and the growth–differentiation balance (GDBH) hypotheses, a higher nutrient availability can lead to plant growth rather than allocation to carbon-based secondary compounds, such as terpenoids, phenolics and flavonoids. On the other hand, a higher nutrient concentration in leaves may translate into higher carbon fixation, higher protein synthesis and higher enzyme activity [68], with consequently more terpene and secondary metabolite production. In this context, several studies have analyzed the relationship between leaf terpenoid concentrations and nutrient availability, although the results are contradictory. Some studies have observed both an increase and decrease in leaf terpenoids with higher leaf N concentrations [57,69], whereas other studies have not found any clear relationship [70]. Similarly, no clear relationship was observed between P and terpenoid concentrations in the leaves of different species [57,69]. In this regard, no significant differences in total and relative leaf terpenoid concentrations among the soil amendment treatments were observed in the present study, although it is generally considered that changes in the absolute amounts of terpenoids occur in response to abiotic factors, including inorganic fertilization [11]. The data in the present study are in line with the findings of chemosystematic studies showing that the constitutive terpenoid and, in particular, monoterpene profiles in healthy mature tissues are under tight genetic control and little influenced by abiotic factors [35,36,37,38]. For example, the use of vermicompost as an organic amendment had no significant effects on the relative contents of 27 out of the 32 terpenoids detected in the essential oils of rosemary plants [71]. Different concentrations of nutrient solutions did not change the chemical composition of the essential oils in rosemary plants growing outside of soil [72]. Nevertheless, the present study shows a positive correlation between both leaf N and P and total terpenoid concentrations (Figure 3). As regards primary metabolism and growth, a positive correlation was found between leaf N and structural carbohydrates. In addition, structural carbohydrate content in the stems was higher in plants grown in fertilized soil than those grown in non-fertilized soils (control conditions). These results reflect the fact that the biomass of plants grown on fertilized soils was higher than the biomass of plants grown on control soils [73]. Our terpenoid results therefore seem to be in contrast with the CNBH and GDBH hypotheses, as N and P availability were related to higher plant growth and higher terpenoid content simultaneously.

We also measured the total amounts of phenolic and flavonoid compounds in the rosemary roots, stems and leaves in order to check if a response of the terpenoid contents to leaf N and P was found for other secondary metabolites. The results showed that the fertilization treatments had a negative effect on the phenolic and flavonoid contents in all the rosemary plant parts. A negative correlation between leaf N and phenolics, and between leaf N and flavonoids was also found. These latter results fit perfectly with the CNBH and GDBH hypotheses, regarding leaf N, and are in line with other previous studies. Langenkämper et al. [74] reported a higher concentration of phenolic compounds in the grains of unfertilized wheat plants than in those of fertilized ones. Moreover, Benard et al. [75] observed that nitrogen deprivation induced a marked increase in chlorogenic acid and rutin levels in tomato leaves.

The results reported here suggest the existence of various mechanisms by which N availability controls the synthesis of the different types of secondary metabolites. Massad et al. [58] also found differences in the effects of available N on the saponin (a triterpenoid) and flavan (a phenolic compound) contents. Whereas the trade-off predicted by the GDBH between metabolite production and growth was present between flavans and biomass, saponins and biomass were positively correlated. These results were obtained under light conditions and, consequently, with moderate carbon resources provided through photosynthesis. The authors suggested that saponin synthesis was more limited by nitrogen (needed for synthesis) than by carbon (needed as a substrate).

It has been also proposed that the phenolic contents fit the CNBH and GDBH hypotheses better because there is a direct N trade-off between growth and the shikimic acid pathway by which phenolic compounds are synthesized [15]. Indeed, restriction of N decreases protein synthesis and thus competition for phenylalanine, a precursor of phenolic compounds. Under low N conditions, the biosynthesis of phenolics can continue, as phenylalanine deamination is the first phase, and the unbound amine group can be recycled to produce more phenylalanine [76]. However, the biosynthesis of terpenoids takes place via the mevalonic pathway and methylerythritol phosphate pathways that do not compete directly with growth for available nitrogen. Massad et al. [58] suggested that saponins and photosynthesis compete for nitrogen before carbon is split between carbon-based secondary metabolites and growth.

The situation for phosphorus was different. Our results showed that neither terpenoids nor phenolics fit the CNBH and GDBH hypotheses on the basis of P availability. Although some studies found that polyphenol concentrations in plants increased [77] or decreased [78] with increasing P availability, it was also suggested that P availability is not important for the production of phenolic compounds, as it is not directly related to the phenylalanine pathway through which proteins and many polyphenols are synthesized [76]. In this context, Wright et al. [79] hypothesized that reduced P availability restricts growth mainly due to the lack of enough P to form cells, so it does not affect protein production capacity and the requirement for phenylalanine. As regards terpenoid concentrations, we suggest that, as happens with N, the production of terpenoids is more limited by P than by C. The terpenoid precursors IPP (isopentenyl diphosphate), DMAPP (dimethylallyl pyrophosphate), GDP (geranyl diphosphate) and FDP (farnesyl diphosphate) contain high-energy phosphate bonds. Moreover, phosphorus is part of the ATP and NADPH molecules, needed for terpenoid synthesis through both the mevalonate (MVA) and the methylerythritol phosphate (MEP) pathways. It was determined that *Quercus coccifera* L. needs 28 moles of NADPH and 40 moles of ATP to synthesize monoterpenoids [48]. Therefore, phosphorus may be a basic element for terpenoid storage.

On this basis, it is reasonable to think that under moderate nutrient soil concentrations, N and P do not limit terpenoid production, because plants may take up enough N and P to fulfill their requirements for growth and terpenoid synthesis [57]. Various soil N and P contents and/or conditions limiting nutrient uptake by plants could thus explain the apparently contradictory results reported in different studies regarding the effect of fertilization on terpenoid contents. Indeed, our results are in contrast with those reported by Ormeño et al. [56] as they did not find any correlation between total leaf isoprenoids in *R. officinalis* and soil N and P (though they did not show N or P contents in leaves). On the contrary, the different nutrient concentrations influenced the content and yield of the essential oils in the rosemary plants grown outside soil [72]. Other studies regarding the fertilization’s effects on terpenoid content in storing species have also led to different results. Blanch et al. [57] found a positive effect of P fertilization on terpenoid contents in *P. halepensis* under drought conditions, whereas Ormeño et al. [56] reported a positive correlation of terpenoid content with soil N and P in the same species. However, it is noticeable that, in the first case, no significant differences in leaf P were found between the control and P-fertilized *P. halepensis* plants.

Finally, it is interesting to note that not all the terpenoids studied changed as a function of leaf N or P, suggesting that their production depends to varying degrees on N and P availability. Only β-pinene, myrcene, limonene and terpineol were positively correlated with leaf N, whereas only α-pinene and sesquiterpenes were correlated with leaf P. In this regard, Chrysargyris et al. [80] showed how different phosphorus application rates in soil altered the amount of carvone, β-caryophyllene, β-myrcene and sabinene in *Mentha spicata* leaves but did not change the amount of other terpenoids. In *Salvia officinalis*, it was also shown how the percentage of β-pinene increased with increasing N levels and how interactive effects between N and P treatments altered the amounts of both α- and β-thujones [81]. This inconsistency in the effects of leaf N and leaf P on the production of different compounds may be related to the genetic control by *R. officinalis* of terpene production. Indeed, terpenoid biosynthesis is known to be under strong genetic control [82,83], although some degrees of phenotypic plasticity can be observed in terpene production as a response to abiotic factors [84] and, in our case, to soil fertilization. Abiotic factor-induced changes in the concentrations of individual secondary metabolites of the same compound class were found for pyrrolizidine alkaloids in *Senecio* (Asteraceae) [85], for phenylpropanoid compounds in tobacco [86] and for phenolics in *Trifolium pratense* [87].

An understanding of how fertilization affects the production of different types of secondary plant metabolite contents may contribute to the comprehension of how plant defense mechanisms can be driven by nutrient availability. In this context, plant terpenoids play the primary ecological role of chemical defense against the attacks of pests and disease [36,88], and leaf terpenoids have been shown to be related to plant thermotolerance and protection against drought and oxidative stresses [1,8]. Moreover, terpenoids also have a role in plant–plant and plant–animal communication [30,89]. Therefore, a higher terpenoid content, as a result of fertilization, is expected to help plants to adapt to abiotic stress conditions and to alter the ecological interaction of plants with the biotic environment [3,4,5,6,7], with consequences for ecosystem functioning. On the other hand, this information could be valuable for growing plants with a greater yield of essential oils and high amounts of biologically active compounds to be used in the food, pharmaceutical and chemical industries and, not least, for the recently discussed potential use of these compounds as natural products with antimicrobial effects, reducing the risk of resistance [90].

## 5. Conclusions

Though several studies have already described how phenolic compounds fit the CNBH and GDBH hypotheses with regard to N availability, contradictory results were found with terpenoid compounds. The results reported here provide evidence of the simultaneous effects of fertilization and nutrient availability on different kinds of carbon-based secondary metabolites in *R. officinalis* plants. Two different control mechanisms regarding terpenoid and phenolic/flavonoid production under fertilization conditions are highlighted. The response of terpenoid production to fertilization was ascribable to leaf N availability, essential for protein synthesis and terpene synthase activity, and to leaf P, required as a component of terpenoid precursors (IPP and GDP) and the ATP and NADPH molecules, needed for terpenoid synthesis. Conversely, the fertilization’s effects on phenolics production was a direct trade-off, based on the available nitrogen, between growth (protein production) and the shikimic acid pathway by which phenolic compounds are synthesized. Finally, different responses of single terpenoids to leaf N or P contents were observed. The behavior of each single terpenoid with regard to nutrient availability was probably the consequence of interactions between genetic and nutritional factors in the regulation of *R. officinalis* plant terpenoid production.

## Figures and Tables

**Table 1:** Leaf and stem concentrations of total N (%), P (g kg^−1^ d.m.) and K (g kg^−1^d.m.) in *Rosmarinus officinalis* plants for the fertilization treatments: Control, InOrg, CS_Low_, CS_High_, PS_Low_ and PS_High_. Mean values ± standard errors (*n* = 3) are shown. Different letters indicate significant differences among fertilization treatments (*p* < 0.05). Asterisks indicate significant differences between leaf and stem values (*p* < 0.05).

Plant Part	Treatment	Total N (%)	P (g kg^−1^)	K (g kg^−1^)
**Stem**	Control	0.69 a* ± 0.06	2.04 a ± 0.26	16.40 b ± 0.04
	InOrg	0.75 a* ± 0.02	2.17 a ± 0.06	14.86 ab ± 1.71
	CS_Low_	0.77 ab* ± 0.13	2.04 a ± 0.01	13.80 a ± 0.24
	CS_High_	0.99 ab* ±0.10	2.30 a ± 0.33	16.61 ab ± 1.57
	PS_Low_	0.84 ab* ± 0.14	2.36 a ± 0.39	15.68 ab ± 0.63
	PS_High_	1.11 b* ± 0.05	2.64 a ± 0.05	19.00 c*± 1.53
**Leaf**	Control	1.72 ab ± 0.16	2.90 a ± 0.01	15.95 ab* ± 0.11
	InOrg	1.71 a ± 0.05	3.03 a* ± 0.06	16.99 bc ± 0.57
	CS_Low_	1.82 ab ± 0.20	2.03 a ± 0.37	16.87 c*± 0.27
	CS_High_	2.14 b ±0.11	3.20 a ± 0.09	16.96 abc ± 1.01
	PS_Low_	1.96 ab ± 0.13	2.36 a ± 0.35	16.24 abc ± 0.41
	PS_High_	2.36 b ± 0.11	3.55 b* ± 0.05	15.42 a ± 0.33

PS or CS: anaerobic digestate-based pig or cattle compost. Doses: Low, 30 t ha^−1^; High, 60 t ha^−1^. InOrg: inorganic NPK fertilizer with the proportions of 100:60:73.

**Table 2:** Relative amounts of terpenoids (% of the total) in *Rosmarinus officinalis* leaves for the fertilization treatments: Control, InOrg, CS_Low_, CS_High_, PS_Low_ and PS_High_. Data reported as mean values ± standard errors (*n* = 3) are shown.

	Control	InOrg	CS_Low_	CS_High_	PS_Low_	PS_High_
(+)-α-pinene	39.6 ± 1.0	34.9 ± 0.6	35.7 ± 1.5	35.4 ± 0.8	37.8 ± 1.2	35.1 ± 1.6
camphene	8.3 ± 0.3	7.5 ± 0.3	8.0 ± 0.5	8.4 ± 0.5	7.6 ± 0.7	9.0 ± 0.2
unknown 5	0.4 ± 0.1	0.4 ± 0	0.4 ± 0	0.4 ± 0.1	0.5 ± 0	0.6 ± 0
sabinene	0.4 ± 0.1	0.3 ± 0	0.4 ± 0	0.3 ± 0.1	0.3 ± 0	0.3 ± 0
(+)-β-pinene	3.2 ± 0.3	3.0 ± 0.3	3.3 ± 0.2	3.1 ± 0.5	3.7 ± 0.4	4.2 ± 0.3
myrcene	3.6 ± 0.1	3.3 ± 0.1	3.6 ± 0.1	3.4 ± 0.1	3.6 ± 0.1	3.5 ± 0.2
limonene	4.4 ± 0.2	4.2 ± 0.1	4.3 ± 0.1	4.3 ± 0.1	4.2 ± 0.1	4.1 ± 0.2
p-cymene	2.7 ± 0.2	2.8 ± 0.3	2.4 ± 0.2	2.4 ± 0.3	1.9 ± 0.4	2.3 ± 0.5
cineole	7.7 ± 0.3	7.9 ± 0.2	7.8 ± 0.2	7.9 ± 0.3	8.4 ± 0.2	8.5 ± 0.3
γ-terpinene	0.5 ± 0	0.5 ± 0	0.5 ± 0	0.5 ± 0	0.7 ± 0	0.5 ± 0
terpinolene	0.6 ± 0	0.8 ± 0.1	0.7 ± 0	0.6 ± 0.1	0.6 ± 0	0.7 ± 0.1
linalool	1.1 ± 0.2	1.2 ± 0.1	1.1 ± 0	1.2 ± 0.1	1.1 ± 0.2	1.0 ± 0.1
camphor	8.9 ± 0.6	9.1 ± 0.3	9.1 ± 0.4	9.6 ± 0.5	9.0 ± 0.6	9.9 ± 0.4
terpinen-4-ol	0.4 ± 0	0.5 ± 0	0.5 ± 0	0.5 ± 0	0.5 ± 0.1	0.5 ± 0
unknown 30	1.0 ± 0	1.2 ± 0	1.2 ± 0	1.2 ± 0.1	1.1 ± 0.1	1.3 ± 0.1
borneol	2.3 ± 0.5	2.4 ± 0.3	2.7 ± 0.2	3.6 ± 0.5	1.9 ± 0.2	2.2 ± 0.3
bornylacetate	0.6 ± 0.2	0.9 ± 0	1.0 ± 0	0.9 ± 0.1	0.9 ± 0.1	0.8 ± 0.1
verbenone	11.3 ± 2.9	15.4 ± 0.7	13.6 ± 2.8	12.7 ± 2.6	13.6 ± 2.8	11.7 ± 3
geraniol	0.3 ± 0.1	0.5 ± 0	0.4 ± 0.1	0.4 ± 0.1	0.4 ± 0.1	0.4 ± 0.1
unknown 37	0.4 ± 0.1	0.6 ± 0	0.5 ± 0.1	0.5 ± 0.2	0.4 ± 0.1	0.6 ± 0
β-caryophyllene	2.0 ± 0.6	2.2 ± 0.4	1.4 ± 0.5	2.0 ± 0.4	1.2 ± 0.4	2.1 ± 0.4
caryophyllene oxide	0.3 ± 0.1	0.3 ± 0.1	0.3 ± 0.1	0.3 ± 0.1	0.2 ± 0.1	0.4 ± 0.1

PS or CS: anaerobic digestate-based pig or cattle compost. Doses: Low, 30 t ha^−1^; High, 60 t ha^−1^. InOrg: inorganic NPK fertilizer with the proportions of 100:60:73.

**Table 3:** Leaf and stem sugar contents from structural carbohydrates (g 100 g^−1^ d.m.) in *Rosmarinus officinalis* leaves for the fertilization treatments: Control, InOrg, CS_Low_, CS_High_, PS_Low_ and PS_High_. Data are reported as mean values ± standard errors (*n* = 3).

Plant Part	Treatment	Glucose	Arabinose	Galactose	Mannose	Xylose	Rhamnose	Fucose
**Leaf**	Control	8.18 ± 0.25	3.32 ± 0.26	2.17 ± 0.06	1.12 ± 0.07	1.05 ± 0.05	0.15 ± 0.05	<0.1
	InOrg	8.06 ± 0.89	3.16 ± 0.29	2.23 ± 0.29	1.20 ± 0.17	1.03 ± 0.20	0.55 ± 0.25	<0.1
	CS_Low_	11.05 ± 1.16	3.17 ± 0.20	2.32 ± 0.31	1.47 ± 0.20	1.15 ± 0.13	0.20 ± 0.00	<0.1
	CS_High_	10.45 ± 0.70	3.25 ± 0.09	2.27 ± 0.09	1.52 ± 0.07	1.17 ± 0.06	0.20 ± 0.00	<0.1
	PS_Low_	8.87 ± 0.30	3.67 ± 0.33	2.50 ± 0.25	1.37 ± 0.13	1.05 ± 0.05	0.20 ± 0.10	<0.1
	PS_High_	8.62 ± 0.38	3.42 ± 0.22	2.27 ±0.13	1.32 ± 0.07	1.15 ± 0.09	0.20 ± 0.10	<0.1
**Stem**	Control	21.00 ±0.70	1.50 ± 0.09	1.00 ± 0.10	0.85 ± 0.03	0.85 ± 0.09	0.15 ± 0.05	<0.1
	InOrg	21.40 ± 0.80	1.90 ± 0.11	1.20 ± 0.01	1.40 ± 0.06	1.22 ± 0.06	0.25 ± 0.05	<0.1
	CS_Low_	21.80 ± 0.30	1.90 ± 0.33	1.50 ± 0.11	1.35 ± 0.14	1.22 ± 0.06	0.30 ± 0.10	<0.1
	CS_High_	21.80 ± 0.30	2.15 ± 0.04	1.40 ± 0.02	1.70 ± 0.01	1.34 ± 0.04	0.40 ± 0.00	<0.1
	PS_Low_	20.65 ± 2.35	2.15 ± 0.25	1.40 ± 0.23	1.40 ± 0.11	1.09 ± 0.05	0.30 ± 0.10	<0.1
	PS_High_	20.10 ±0.02	1.90 ± 0.03	1.50 ± 0.20	1.40 ± 0.06	1.18 ± 0.04	0.35 ± 0.05	<0.1

PS or CS: anaerobic digestate-based pig or cattle compost. Doses: Low, 30 t ha^−1^; High, 60 t ha^−1^. InOrg: inorganic NPK fertilizer with the proportions of 100:60:73.
